# Preconcentration of trace amount Cu(II) by solid-phase extraction method using activated carbon-based ion-imprinted sorbent

**DOI:** 10.3906/kim-2110-41

**Published:** 2021-12-24

**Authors:** Kübra TURAN, Rukiye SAYGILI CANLIDİNÇ, Orhan Murat KALFA

**Affiliations:** Department of Chemistry, Science and Art Faculty, Kütahya Dumlupınar University, Kütahya, Turkey

**Keywords:** Copper, preconcentration, surface-ion imprinting technique, selectivity, flame atomic absorption spectrometry (FAAS)

## Abstract

In this study, preconcentration conditions of trace amounts of copper ions were investigated with solid-phase extraction (SPE) method by synthesizing activated carbon-based ion-imprinted sorbent (Cu(II)-IAC) with a novel and selective approach. Flame atomic absorption spectrometry (FAAS) was used for the determination of metal ions concentrations. For the characterization of the sorbents, scanning electron microscopy, energy dispersive X-ray (SEM/EDX) analysis, and Fourier transform infrared spectroscopy (FTIR) were used. Optimum conditions for the SPE procedure, various parameters such as pH value, eluent type and concentration, sample volume, sample flow rate, adsorption capacity, and selectivity were studied. The adsorption isotherm was analyzed by Freundlich and Langmuir isotherm, and the maximum adsorption capacity was found to be 142.9 and 312.5 mg/g for activated carbon-based nonimprinted (Cu(II)-non-IAC) and Cu(II)-IAC sorbents, respectively from the Langmuir isotherm. Limit of determination (LOD) and limit of quantification (LOQ) values were found to be 0.038 and 0.113 μg/L, respectively for Cu(II)-IAC sorbent, and the results were compared with the literature. The accuracy and validity of the proposed method were evaluated by the determination of Cu(II) ions from tap water samples and certified reference materials (CRMs) (soft drinking water ERML-CA021e and NIST 1643e) analysis. Good and quantitative recoveries were obtained for the spiked analysis.

## 1. Introduction

Heavy metal pollution is one of the biggest environmental problems worldwide. These metals are released into the environment from many different sources such as industry, wastewater, agricultural waste, batteries, automotive, etc. [1–5]. Among these sources, water is one of the important exposure sources of heavy metals for the human body. One of these heavy metals is copper (Cu), which is an essential element class for the human body, and its excess can be poisonous when it’s released into environment via machinery manufacturing, metallurgy, smelting, electronics, and pharmaceutical industries [6–9]. The maximum permissible limit for Cu(II) ions in drinking water as set by the United States Environmental Protection Agency (US EPA) is 1.3 mg/L [10]. Therefore, its determination is extremely important even in trace amounts. So, it is a scientific research area for analytical chemists in order to check the level of contamination of Cu (II) in the environment and develop new methods to eliminate the pollution from copper.

Flame atomic absorption spectroscopy (FAAS) is one of the most used methods for metal determination, but, especially in environmental samples, a pretreatment/separation is required due to the complex matrices and the usually low concentration levels [11–13]. For this purpose, preconcentration techniques are most widely used. Several techniques such as solid-phase extraction (SPE) [14–16], liquid-liquid extraction (LLE) [17,18] and coprecipitation [2,19] have been utilized for the separation and determination of metals with high sensitivity. However, the applicability of these procedures may not always be suitable due to the time-consuming, unsatisfactory preconcentration factor, low capacity, sensitivity, and the use of organic solvents. Among these, SPE is the most suitable technique due to its high recovery, good capacity, and high preconcentration factor. The most important parameter of this technique is the proper sorbent selection [20]. Because the success of the analytical method depends on the sorbent type for preconcentration procedures. Many different types of sorbents can be used, but one of the most important properties is the selectivity and capacity of the sorbent. Recently, for this purpose, imprinted sorbents have attracted great interest.

Activated carbon (AC) has been one of the important sorbents used in various studies due to its large specific surface area, high porosity, stable chemical properties, and low cost [21,22]. The physical and chemical structure of carbon can be changed by the activation process. For this purpose, different activation techniques can be used. Chemical activation is more advantageous than physical activation [22,23]. In the activation process, since the amount of active site and the pore structures of AC are determined by activating agents, they play a crucial role in the structure of carbon materials [24]. Acid treatment of carbon is generally used to oxidize carbon surface because it increases the acidic property, removes elements, and improves the hydrophilic structure of the surface [25]. Many acidic agents such as HCl, HNO_3_, H_2_SO_4_ are used for this purpose. Modification with HNO_3_ increases the amount of carbonyl, carboxyl, phenolic, and lactone groups that give the surface an acidic character. As these groups increase on the surface, the electronegativity of the sorbent increases. Metal ions tend to form metal complexes with negatively charged acid groups. In other words, surface polarization decreases, and, as a result, the affinity of the sorbent to heavy metals and hence the adsorption capacity of activated carbon increases [23,26].

Generally, surface modification of the AC is carried out after the activation step [26]. The modification changes the amount and diversity of surface functional groups, which are highly influential on the adsorption properties of activated carbons [23]. The surface of AC has a relatively limited number of functional groups. Several agents are used for the modification of the active carbon. Amine groups are widely used for the functionalization of the sorbent surface. Amine-modified materials have already shown high affinity and capacity towards heavy metals [27–31].

The imprinting technique is used to create spaces with specific selective binding sites specific to a particular molecule or ion. This technology allows for the preparation of synthetic sorbents for the target molecule or ion, which are formed by using the template molecule or ion, having specific binding sites. The prepared imprinted sorbents are capable of reconnecting the template molecule or ion with a key-lock type interaction. The molecule, ion, or dummy molecule can be used as a target molecule. When using ions as an imprinting template, the method is called ion imprinting [32,33].

The surface ion imprinting technique (SIIT) can be used as a basic method for identifying certain chemical species. SIIT is one of the important synthesis methods for preparing ion-imprinted material, which has superior advantages over conventional techniques. With this technique, sorbents could be synthesized by simple and easy preparation acquire extraordinary properties such as high adsorption capacity, accessibility, high selectivity, fast adsorption kinetics, preparation, low mass transfer resistance, and complete removal of target ions. Also, SIIT has recognition gaps found at or near the polymer surface [33–41]. Therefore, sorbents obtained by SIIT are practical and can be easily used for adsorption and in the SPE technique for preconcentration [42–51].

A selective active carbon based Cu(II) ion-imprinted sorbent (Cu(II)-IAC) was prepared through the ion imprinting technique and used for the Cu(II) preconcentration in this study. For comparison, the nonimprinted sorbent (Cu(II)-non-IAC) was synthesized without metal ions by the same technique. Determination of metal ions was carried out with FAAS. When compared to literature, better values were obtained such as column regeneration, preconcentration factor, the limit of detection, and adsorption capacity. In this point of view, a novel, selective adsorbent has been introduced to the literature with an effective and easy method for the preconcentration/determination of copper.

## 2. Materials and methods

### 2. 1 Chemicals

All chemicals were of analytical grade reagent. Atomic spectroscopy standard (Perkin Elmer, USA) stock Cu(II) solution (1000 μg/mL) was used and diluted to the desired value. All required metal solutions were obtained from their respective nitrate salts and ICP standards as Ni, Mn, Zn, Cd, Pb, Co, Cr, and Cu(NO_3_)_2_ (Merck, Germany). Activated carbon (Sigma-Aldrich, USA), hydrazine hydrate (Merck, Germany), HNO_3_ (65%, Merck, Germany), NaOH (Merck, Germany), HCl (37%, Merck, Germany), ethanol (Merck, Germany), and N-methoxymethyl melamine chemical basis a crosslinker (RUCO-COAT FX 8000, Rudolf Duraner, Bursa, Turkey), ethylendinitrilo tetraacetic acid, disodium salt dihydrate (Titriplex III) (Merck, Germany) were used in the experiment. NIST 1643e and soft drinking water (certified reference material) obtained from National Institute of Standard and Technology, (Gaithersburg, USA).

### 2.2 Instrumentation

A PinAAcle 900T model (Perkin Elmer, USA) atomic absorption spectrometer (FAAS) with air/acetylene flame was used to determine metal ions concentration. Wavelength = 324.75 nm, slit width = 0.7 nm, desired current = 30 mA, acetylene/air flow rate = 2.5/10 mL/min. All separations were carried out on a (0.8 mm × 15 cm) homemade glass column. All pH measurements were done with Seven Compact model (Mettler Toledo, Switzerland) pH meter. Longerpump BT100-1L model peristaltic pump (China) was used. For the characterization, FT-IR (Bruker-Alpha, Germany), scanning electron microscopy, and energy dispersive X-ray (SEM/EDX) (FEI-Thermo Fisher Scientific, USA) techniques were used. Thermal analysis studies were performed with SII EXSTAR 6000 TG/DTA 6300 device and TG, DTG, and DTA curves were taken at the same time. In all experiments, Millipore Elix Essential 10 ultrapure system (18 W/cm), (Merck Germany) was used for ultrapure water.

### 2.3 Preparation of the Cu(II) ion-imprinted and non-imprinted sorbents

The structural properties and surface chemistry of the sorbents have a remarkable impression on the adsorption performance. The activation operation changes the structure of carbon materials. Treatment with HNO_3_ of carbon is generally used to oxidize carbon surface because it increases the acidic property. Metal ions tend to form metal complexes with negatively charged acid groups, hence the adsorption capacity of activated carbon increases. Amine-modified materials have been shown to have high affinity and capacity towards heavy metals. For this purpose, AC used for the synthesis of sorbents was first activated with acid, and then the imprinting process was carried out with a metal-amine complex. Amine was used here due to its high affinity for heavy metals.

Synthesis of the Cu(II) ion-imprinted and nonimprinted sorbents were performed in two steps [29,32]. The steps of the synthesis procedure are as follows:

1. ***Activation procedure:*** A total of 5 g of active carbon (AC) was treated for 24 h at 55°C with 100 mL 10% (v/v) HCl, then it was washed and dried overnight. Afterward, the dried solid was taken in 150 mL, 35% (v/v) HNO_3_ and stirred for 24 h at 55°C for the activation process then filtered and washed with ultrapure water until neutral pH value is obtained. Lastly, the last solid was dried at 80 °C in an oven. The AC that opened active ends was named AC-COOH.
2. ***Synthesis of Cu(II)-IAC and Cu(II)-non-IAC:*** 4 g of Cu(NO_3_)_2_.3H_2_O was dissolved in 100 mL of ethanol. A total of 10 mL of hydrazine hydrate solution was added and mixed. Afterward, 10 g of AC-COOH and 10 mL of cross-linker were added into the mixture, respectively. This mixture was stirred at 75 °C for 72 h at 500 rpm. Then, the mixture was evaporated, and the solid was dried at 80 °C. 0.1 mol/L Titriplex III solution was used to remove Cu(II) ions from the sorbent surface. This solution was filtered, and the last product was washed with diluted HNO_3_, ultrapure water in abundance, and dried at 100°C in an oven. The synthesis of nonimprinted sorbent Cu(II)-non-IAC was performed with the same procedure without adding Cu(II) ions.

### 2.4 Solid phase extraction procedure

Each sorbent was weighed as 0.100 g and were placed in a 0.8 mm × 15.0 cm diameter a glass column. Glass cotton was placed above and below the sorbent to prevena t sorbent missing from the column. Sample and elution solutions were passed through the column by peristaltic pump. Firstly, for conditioning, the column was treated with 25 mL of 1.0 mol/L HNO_3_ solution and then washed with ultrapure water. Diluted HCl or NaOH solutions were used for pH adjustment. Then, 25 mL of 0.5 mg/L Cu(II) solutions, which were adjusted to working pH, were passed through the column and treated with eluent solution. The column was washed with eluent and ultrapure water after each use and kept in ultrapure water until for further experiment.

### 2.5 Batch method

A total of 25 mL of sample solutions containing Cu(II) ion with concentrations between 25 and 1500 μg/mL were added into beakers containing 15 mg sorbent, and the pH vaue of the mixture was adjusted to optimum pH value. The solutions were shaken for two hours at 500 rpm at room temperature. After filtration, a suitable dilution was made for each solution, and Cu(II) ions were determined by FAAS.

### 2.6 Selectivity and adsorption capacity

#### Adsorption capacity

Since the system needs to have a balance in order to determine the adsorption capacity, the batch system (Section 2.5) was used. The adsorption capacity of the sorbent determines the effectiveness of the sorbent, which removes the contaminant. The amount of material adsorbed on the sorbent is calculated according to the concentration remaining in the solution. Adsorption isotherms help to predict the adsorption mechanism, interactions between the metal, and the sorbent during the adsorption process at a constant temperature and a constant solution pH value and include mathematical equations to calculate some parameters such as adsorption capacity. Mathematical equations and optimal model provide information about the adsorption mechanism and surface properties of the sorbent and the adsorption models have been developed to predict monolayer/multilayer or homogeneous/heterogeneous adsorption mechanism. To explain the adsorption behavior, the linear regression constant (R^2^) value helps to find out which adsorption isotherm is suitable [52,53]. The most widely used adsorption isotherms are the Langmuir and Freundlich adsorption isotherms that calculate the maximum adsorption capacity (Q_m_) of the sorbents.

Langmuir adsorption isotherm [54] is the most widely used adsorption isotherm. In this model, (1) the surface is homogeneous, (2) adsorption is a monolayer process, (3) no lateral interaction between adsorbed molecules, and (4) the adsorption is reversible [53,55]. The mathematical representation of the Langmuir adsorption isotherm is expressed as follows:


(1) $$\frac{C_{e}}{q_{e}}=\frac{C_{e}}{q_{m}}+\frac{1}{q_{m}\;K_{L}}$$

Here, Q_e_ (mg/g) signifies the amount of metal (mg) adsorbed per unit of sorbent (g), C_e_ (mg/g) signifies the amount of metal remains in the solution at equilibrium, Q_m_ (mg/g) signifies the maximum adsorption capacity, and K_L_ (L/mg) is the Langmuir isotherm constant. The value of K_L_ correlates with the variation in surface characteristics of the sorbent, such as specific surface area and porosity. The Langmuir isotherm graph is plotted by C_e_/q_e_ (Y-axis) against C_e_ (X-axis), and a straight line is observed with intercept 1/(q_m_.K_L_) and slope 1/q_m_ values. The regression constant (R^2^) value from the graph shows the applicability of this model. Adsorption behavior of a number of heavy metal ions, dyes, and other pollutants can be explained with the Langmuir adsorption isotherm system [56].

Freundlich isotherm [57] can describe nonideal, multilayer, reversible adsorption at a heterogeneous surface. The isotherm also assumed that all the adsorption sites have different binding energies [55]. The mathematical equations of the Freundlich adsorption isotherm can be written as follows:


(2) $$q_{e}=K_{F}.{C_{e}}^{\frac{1}{n}}$$

(3) $$\text{log }(q_{e})=logK_{F}+\left(\frac{1}{n}\right).logC_{e}$$

where q_e_ represents the amount of metal (mg/g) adsorbed onto the sorbent at equilibrium, C_e_ signifies the amount of metal (mg/g) remains in the solution at equilibrium, K_F_ is the adsorption capacity of sorbent (mg/g), and n represents the adsorption intensity. The Freundlich adsorption graph is plotted log q_e_ (Y-axis) against log C_e_ (X-axis) to calculate K_F_ and n using intercept and slope, respectively. When the graph is drawn, a smooth line with a slope of 1/n and a intercept of log K_F_ is obtained [57,58].

#### Selectivity

Competitive adsorption experiments were carried out in batch system in aqueous solution to determine the selectivity of determination elements. For this purpose, competitor ions at constant concentrations were added to the medium. A total of 25 mL of sample solutions were added to beakers containing an appropriate amount of sorbent, and the pH value was adjusted to the most appropriate value determined experimentally. The solutions were stirred for two hours at 500 rpm at room temperature. During the mixing process, the pH was continuously controlled, and the pH was kept at the desired value. The determinations of the filtered solutions were made by FAAS. These experiments are performed separately for Cu(II)-IAC and Cu(II)-non-IAC sorbents. The selectivity coefficients can be calculated as follows:


(4) $${\text{K}}_{\text{d}}=\left(\frac{\left({\text{C}}_{\text{i}}-{\text{C}}_{\text{f}}\right)}{{\text{C}}_{\text{f}}}\right).\frac{\text{V}}{\text{m}}$$

(5) $$\text{k}=\frac{{\text{K}}_{\text{d}}\;({\text{Cu}}^{2+})}{{\text{K}}_{\text{d}}\;({\text{X}}^{\text{n}+})}$$

(6) $${\text{k}}^{\prime }=\frac{{\text{k}}_{\text{imprinted}}}{{\text{k}}_{\text{non}-\text{imprinted}}}$$

K_d_ is the distribution coefficient; C_i_ is the concentration of the analyte solution before adsorption (μg/mL); C_f_ is the concentration of the Cu(II) solution after adsorption (μg/mL); k is the selectivity coefficient; k’ is the relative selectivity coefficient.

### 2.7 Charactarization

In order to determine surface properties and structure of the sorbents, the functional groups are important characteristics. FT-IR spectroscopy is an important technique that can give useful information about the material structures. FT-IR analyses were performed for the determination of the chemical bond structure of the synthesized sorbents. SEM images were taken at certain stages of the synthesis in order to examine the porous structure of the synthesized material and to determine the morphology of the sorbents. The changes in the structure of the sorbents obtained by applying heat treatment in the presence of a dry air environment were examined in the graphs drawn as thermogravimetric analysis (TGA), differential thermogravimetric (DTG), and differential thermal analysis (DTA) against temperature [46].

### 2.8 Optimization of method

#### Effect of pH value

pH value is one of the important parameters that directly affect the adsorption capacity. The effect of pH value on adsorption capacity can be explained as the competition of metal ions with hydronium ions for binding sites. At lower pH values, the total surface charge is positive. In these pH values, the presence of H^+^ ions in higher amounts creates competition for the bonding sites on the surface. As it is understood from the low metal adsorption capacity, the dominant side in this race is H^+^ ions. However, as the pH value increases, the total surface charge is negative, so hydronium ions begin to separate from the surface. Thus, positively charged metal ions are attached to free binding sites. In this study, the recovery of Cu(II) ions was investigated in the range of pH = 2–8. The desired pH value was adjusted with diluted solutions of HCl or NaOH. At the end of the preconcentration process, metal concentration was determined by FAAS.

#### Effect of eluent type and concentration

The type and concentration of eluents in the preconcentration parameters have a great effect on analytical performances and recovery. The metal ions adsorbed on the sorbent are eluted with several organic or inorganic solvents [20]. In this study, different concentrations of HCl and HNO_3_ acids were used as eluent.

#### Effect of sample flow rate

Sample flow rate is important for SPE because the binding of metal ions on the sorbent in the column can be only achieved with the appropriate flow rate by a peristaltic pump. In order to achieve a high recovery efficiency, it is important to choose the most suitable flow rate. For this purpose, flow rates between 12 and 96 rpm were investigated.

#### Effect of sample volume

In order to determine the maximum applicable volume of sample solution for the preconcentration process, a series of solutions containing 0.5 mg/L Cu (II) ion and volume ranges of 25–50–100–150–250–500–600–750 mL were prepared, and they passed through the column in optimum conditions. Here, the amount of metal ion was kept constant (12.5 μg) in the solution, and the volume was increased.

## 3. Results and discussion

### 3.1 Characterization of the sorbents

#### FT-IR

**Figure 1:**
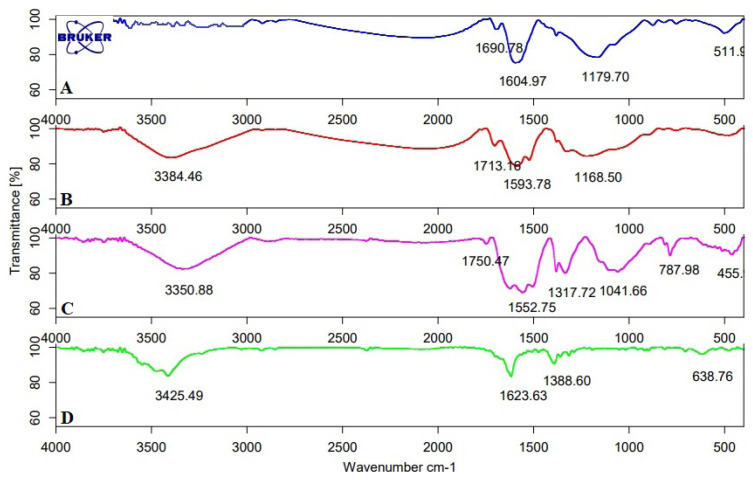
FTIR spectrum of (a) AC, (b) AC-COOH, (c) Cu(II)-IAC and (d) Cu(II)-non-IAC sorbents.

FT-IR spectrum of sorbents was given in Figure 1. AC (Figure 1a) is raw activated carbon. The spectra were approximately very similar for the AC-COOH (Figure 1b); however, they differed from AC, which was a result of the chemical and thermal treatment. Some bands disappeared or weakened during the sorbent preparation including the imprinting and the activation; in particular, the bands located in two regions between 3600 and 2800 cm^−1^ and between 1800 and 800 cm^−1^. The broad-band at about 3000–3500 cm^−1^ showed the presence of -OH stretching vibration carboxylic acid (Figure 1b–d). This broadband was observed after the activation process. -C-H stretching vibration peaks of AC were observed at 2800–3000 cm^–1^, and broad-band around 950–1400 cm^−1^ was due to C-H vibration. The band at 1690 cm^−1^ could be described to C=C stretching vibration. Another strong peak at 1700–1750 cm^−1^ was described to C=O vibrations from carboxylic acids (Figure 1b–d). This band was visible in AC-COOH but not in AC. These results indicated that the activation of AC was performed successfully. The weaker bands between 750 and 511 cm^−1^ were described to aromatic structures. The very strong band at 1000–1150 cm^−1^ represented C-O stretching vibrations. The absorption band appeared at 1560 cm^−1^ and 789 cm^−1^ corresponded to the N-H vibrations. The absorption peak observed at 1317 cm^−1^ corresponded to the stretching vibration of C-N (Figure 1c,1d) [40]. The presence of C–N stretch band indicated that the amine group was bonded successfully.

#### SEM/EDX

**Figure 2:**
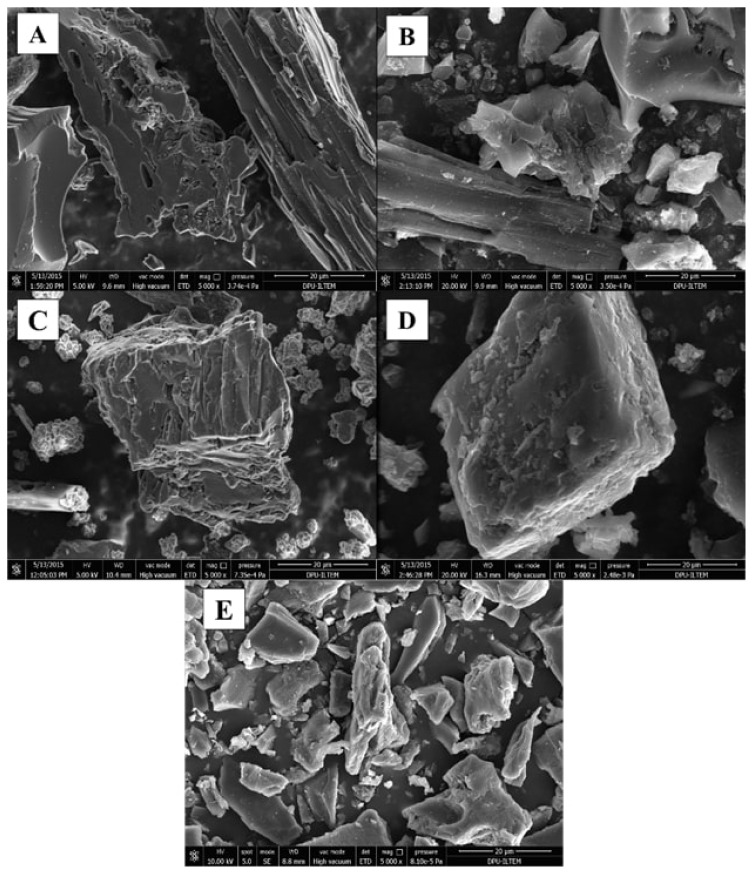
SEM images of (a) AC, (b) AC-COOH, (c) Cu(II)-IAC prior to imprinting, (d) Cu(II)-IAC, and (e) Cu(II)-non-IAC sorbents.

The porous structure of the sorbent could be clearly observed during the synthesis stages (Figure 2**)**. Figure 2b shows that acid treatment was done and opened active ends, and it was named AC-COOH. In Figures 2d and 2e, the surface structure and physical characteristics of Cu(II)-IAC and Cu(II)-non-IAC sorbents were changed, and a high surface area was obtained. Below, SEM images of AC, AC-COOH, Cu(II)-IAC prior to imprinting, Cu(II)-IAC, and Cu(II)-non-IAC were given in Figure 2, respectively. Hereby, it was seen that the imprinting process was performed successfully.

**Figure 3:**
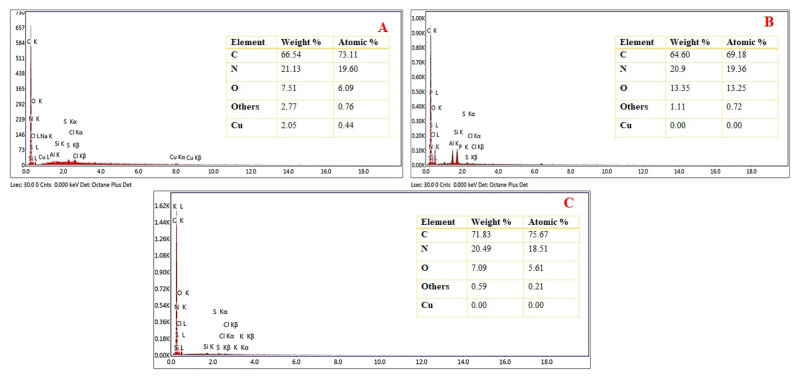
EDX images of (a) Cu(II)-IAC prior to imprinting, (b) Cu(II)-IAC, and (c) Cu(II)-non-IAC sorbents.

The EDX analysis was used to determine the composition of Cu(II)-IAC and confirm that the Cu(II) ion was removed successfully. The EDX spectra of Cu(II)-non-IAC and Cu(II)-IAC before and after removal Cu(II) ions are shown in Figure 3. The results in Figure 3a confirm the presence of C, O, Cu, and others in the structure. As shown in Figure 3b, the peaks of Cu(II) ions was disappeared in the structure of Cu(II)-IAC after removal, indicating that the removal of Cu(II) ions was performed successfully. It also proved that the imprinting process was successfully achieved. In addition, from Figure 3c, Cu(II) peaks were not observed in Cu(II)-non-IAC sorbent because Cu(II) was not added. At the same time, EDX analysis was used to prove the existence of – N groups in Cu(II)-IAC and Cu(II)-non-IAC.

#### TGA

**Figure 4:**
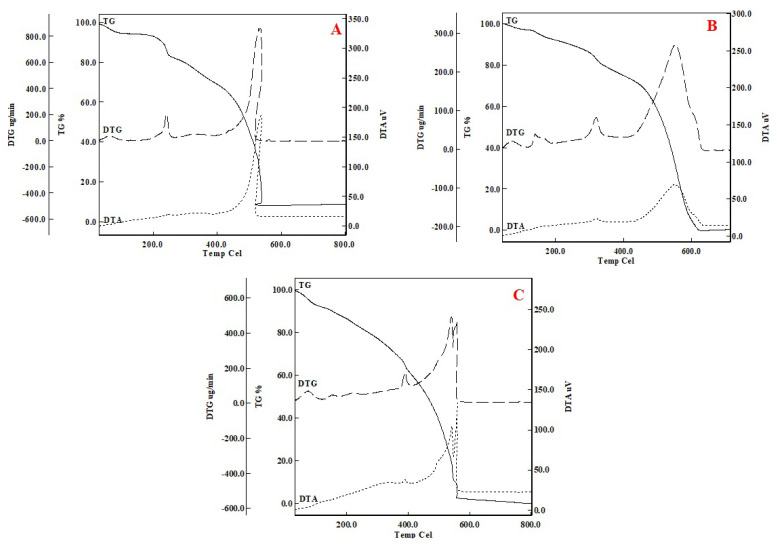
TGA/DTA images of (a) Cu(II)-IAC prior to imprinting, (b) Cu(II)-IAC, and (c) Cu(II)-non-IAC sorbents.

The thermal behaviors of the sorbents before and after imprinting Cu(II)-IAC, and Cu(II)-non-IAC sorbents were investigated under the same conditions using thermal techniques. Thermal curves were given in Figure 4.

TG thermogram showed one main separation step for example. When thermograms were examined, it was observed that there were multiple degradation steps, but the intermediate products were not stable. It was thought that when was heated up to 80, 95 °C for the sorbents Cu(II)-IAC prior to imprinting and Cu (II)-IAC, the hydrating molecule(s) from the structure was removed from the structure accompanied by an exothermic peak (Figure 4a–4b). An endothermic peak at 91 °C was observed in the Cu(II)-non-IAC sorbent (Figure 4c). The endothermic peaks around 100 °C in the graphs were the result of water evaporation. Exothermic peaks could often provide clues about the components of the organic structure depending on the arrangement. DTG maximum peaks observed at 50 °C also confirmed that a group has moved away from the structure. As Figure 4a–4c was shown, decomposition continued up to 550, 620, and 580 °C, respectively. The horizontal curve indicated no deterioration in the sample. It was found by the value read from the TG curve in which Cu(II) metal ion was present in the structure. The final degradation product in Figure 4a (Cu (II)-IAC prior to imprinting) was CuO. It was observed that the CuO% value was 8.2. In Figure 4b curve, the absence of final decomposition products indicated that the metal removal process was successful and that there was no metal in the structure. Likewise, it was clearly observed that there was no metal in the Cu (II)-non-IAC sorbent.

In DTA thermograms, exothermic peaks were generally related to chemical reactions, polymerization, or crystallization processes, and endothermic peaks were related to phase changes, dehydration, reduction, and degradation. In the second step, it was thought that the active carbon and hydrazine structures were removed with an exothermic peak (DTGmax. = 532, 589, and 572 °C) between approximately 90 and 620 °C.

### 3.2 Optimization of method results

#### Effect of pH

**Figure 5:**
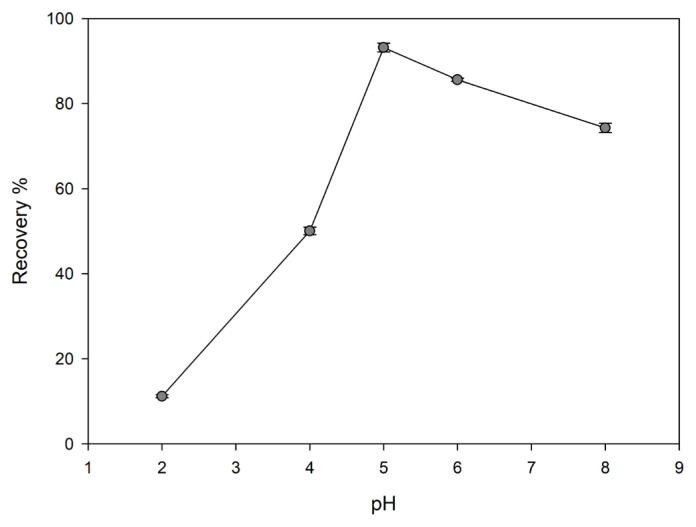
Effect of pH value on the recovery of Cu(II) ions from Cu(II)-IAC sorbent (sorbent dosage: 100 mg, analyte concentration: 0.5 mg/L, flow rate 2 rpm, sample volume: 25 mL, eluent: 0.5 mol/L HNO_3_).

Optimum pH value for the recovery of Cu(II) was detected as described in Section 2.8; Figure 5 shows the changes in the recovery with pH values for Cu(II)-IAC and Cu(II)-non-IAC. The highest recovery was obtained at pH = 5. Thus, the pH = 5 was chosen as the optimum pH value.

#### Effect of eluent type and concentration

Eluent type, concentrations, and recovery efficiency values were given in Table 1. The highest recovery efficiency was obtained with 5 mL of 4 mol/L HNO_3_. So, 4 mol/L HNO_3_ was selected as eluent and was used in subsequent experiments.

#### Effect of sample flow rate

**Figure 6:**
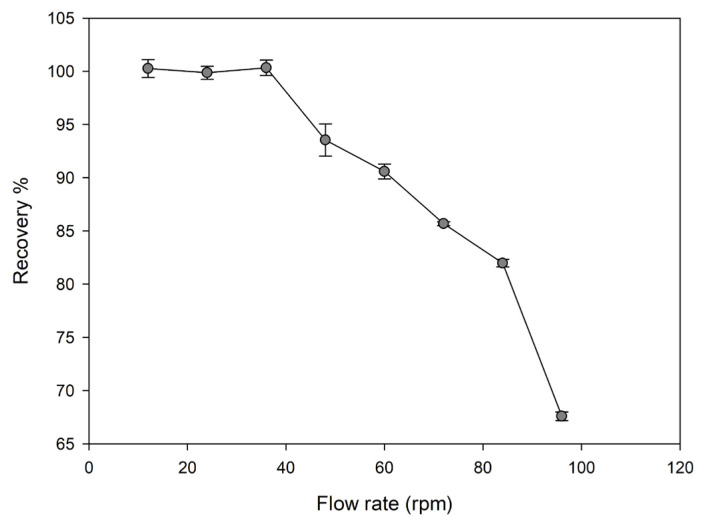
Effect of sample flow rate on the recovery of Cu(II) ions from Cu(II)-IAC sorbent (sorbent dosage: 100 mg, analyte concentration: 0.5 mg/L, pH: 5, sample volume: 25 mL, eluent: 0.5 mol/L HNO_3_).

As given in Figure 6, the results for the recovery of Cu(II) ions on the Cu(II)-IAC were quantitative ( > 95%) up to 48 rpm.

#### Effect of sample volume

**Figure 7:**
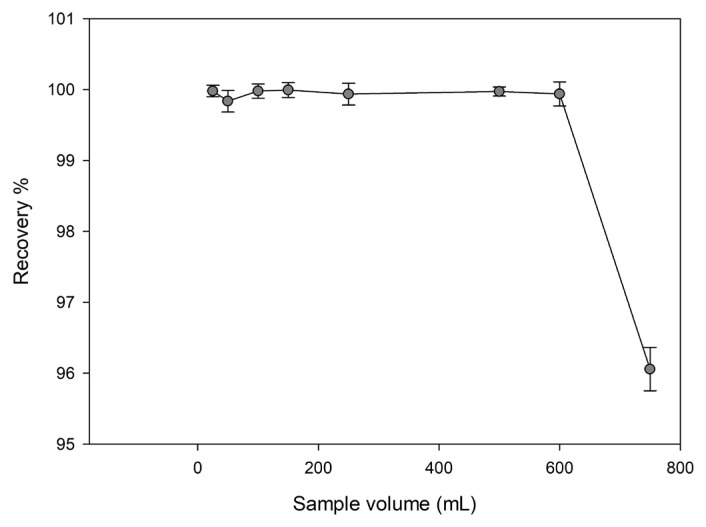
Effect of sample volume on the recovery of Cu(II) ions from Cu(II)-IAC sorbent (sorbent dosage: 100 mg, analyte amount: 12.5 μg, flow rate 48 rpm, eluent: 0.5 mol/L HNO_3_).

The recovery percentage obtained vs. the sample solution volume curve was given in Figure 7. When the obtained results were examined, quantitative results were obtained for samples in the range of 25–750 mL. The highest solution volume applicable with this method was determined as 750 mL. The preconcentration factor (PF) is one of the most important parameters showing the quality of an SPE method. PF was calculated by dividing sample volume by eluent volume. For this study, the PF was found to be as150 (750 mL/5 mL).

#### Selectivity studies

Selectivity is one of the most important parameters for the application of sorbents. Since the synthesized sorbent has ions-specific spaces, they are expected to have high selectivity coefficients. Selectivity studies have been carried out to determine the selectivity coefficients.

As described in Section 2.6, selectivity studies were performed to determine competitive adsorption capability for both Cu(II)-IAC and Cu(II)-non-IAC sorbents in solutions containing different concentrations of metal ion mixture in a batch system. Afterward, the distribution ratios (K_d_), selectivity coefficients (k), and relative selectivity coefficients (k’) for Cu(II) and foreign ions (Mn(II), Ni (II), Zn(II), Cd(II), Pb(II), Co(II), and Cr(III)) were calculated using equations (3–5), respectively. The Cu(II) adsorption capacity of the Cu(II)-IAC was found higher than that for other metal ions. The adsorption capacity of the imprinted Cu(II)-IAC sorbent for Cu(II) ions was also higher compared to the Cu(II)-non-IAC sorbent. As a result, the imprinted sorbent has been determined that be possess a better selectivity coefficient than the nonimprinted sorbent. Selectivity coefficients for all metals and each sorbent were given in Table 2.

In the selectivity results of the sorbents, the ion imprinting effect was clearly observed by comparing them in terms of k’ values. This selectivity effect had been achieved by special spaces for Cu(II) ions formed on the sorbent surface. Therefore, studies that performed with nonimprinted sorbents were not any significant selectivity observed. With this selectivity of the Cu(II)-IAC sorbent to the copper ions, even in the presence of foreign ions in the same matrix, Cu(II) ions could be determined.

#### Adsorption isotherm

**Figure 8:**
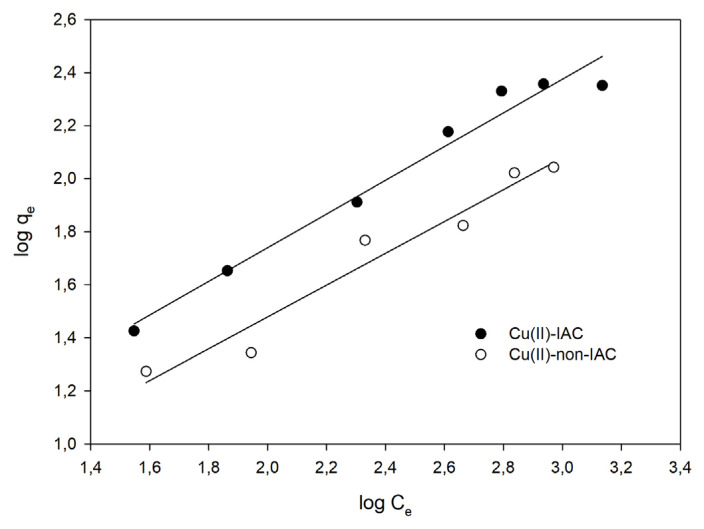
The Freundlich isotherm graph.

**Figure 9:**
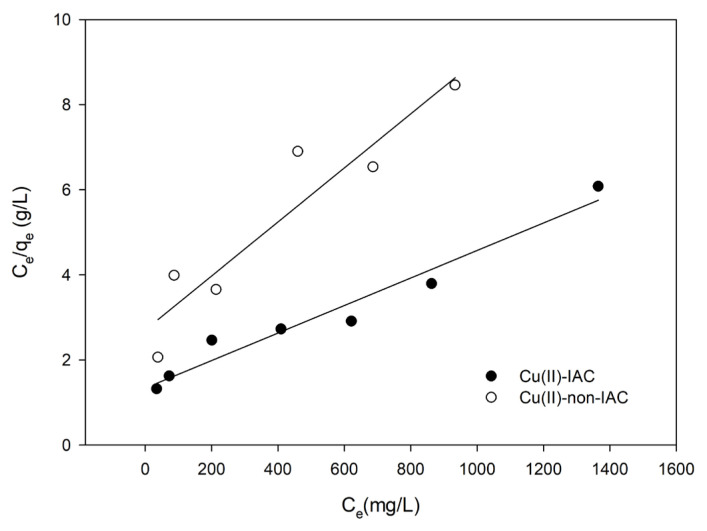
The Langmuir isotherm graph.

The adsorption capacity studies were performed for the imprinted and nonimprinted sorbents separately, as described in Section 2.6. In this way, imprinting had been observed to have an effect on capacity. Langmuir and Freundlich isotherms were plotted (Figure 8 and 9). Langmuir constants of Q_m_ and K_L_; Freundlich constants n and K_F_ were calculated by using the slope and intercepts. Calculated constants were given in Table 3.

From Table 3, n > 1 showed that the adsorption process was favorable. Since R^2^ of the Freundlich isotherm was larger, the adsorption isotherm was suitable for the Freundlich isotherm. The fact that the adsorption conformed to the Freundlich isotherm also indicated that the surface had heterogeneous properties by multilayer adsorption, and adsorption occurred physically [53]. Maximum adsorption capacity and the Langmuir isotherm constants of Cu(II)-IAC and Cu(II)-non-IAC sorbents were calculated as 312.5 mg/g, 0.00239 L/mg, 142.9 mg/g, and 0.00254 L/mg, respectively. The results indicated the success and efficiency of the imprinting process. There was a noticeable increase about 2-fold in the adsorption capacity of the Cu(II)-IAC sorbent when the adsorption capacity was compared.

#### Column regeneration

The column cycle was completed by passing through a glass column the sample solution, eluent, and washing water. The reusability and stability of the sorbent are determined by repeating this process. For this purpose, sample solution, eluent, and washing water were passed through the column filled with the sorbents. The column was kept in water during the period it was not used. As a result of the studies, it was determined that the column regeneration was at least 100 cycles without a significant decrease in the recovery values.

#### Analytical performans

The LOD and LOQ values were calculated in order to determine the analytical performance of the proposed method. For this purpose, in order to obtain the lowest readable signal, 25 mL ultrapure water that found a few drops Cu(II) ions from 0.5 μg/mL solution were added and passed through the Cu(II)-IAC filled column at the optimum conditions. Then ions were eluted with 25 mL of 4 mol/L HNO_3_ from the column without preconcentration. The concentration of Cu(II) ions eluted solution was read 30 times by FAAS.

Instrumental LOD was found after dividing the three times of standard deviation by the slope of the calibration curve (3σb/m). Where σb is the standard deviation of the witness test solution (N = 30), m is the slope of the calibration line. The LOQ value corresponds to approximately 3 times the LOD value. The analytical LOD was calculated by dividing the calculated instrumental LOD value by the preconcentration factor (PF) and the analytical LOQ was calculated by dividing the instrumental LOQ value by the PF [59]. The analytical properties are given in Table 4.

To determine the precision of the method, sample solution, recovery solution, and washing water were passed through the column, which was adjusted to the most suitable conditions for the preconcentration of Cu(II) ions, respectively. This process was repeated 5 times, and the precision of the method was determined by calculating the average recovery efficiency. For the copper ion, the recovery efficiency was 99.6 ± 0.05% at a 95% confidence level. The reproducibility of the results (relative standard deviation < 5%) obtained for the Cu(II) ions is very good and at acceptable levels for trace element determination. In this situation, it can be said that the method has good repeatability. The precision data are given in Table 5.

#### Accuracy and applicability to real samples

In order to test the accuracy of the proposed method, the method should be applied to the certified reference materials. For this purpose, the method was applied to the named “soft drinking water-metals” coded ERMLCA021a and named “simulated freshwater” coded NIST 1643e. 5 mL of the standard reference materials were taken and 1.00 μg/mL Cu(II) spiked into NIST 1643e and each was diluted to 25 mL. After the prepared solutions were adjusted to the appropriate pH value, they passed through the column filled with Cu(II)-IAC under optimum conditions. Elution of Cu(II) ions was performed with 4 mol/L HNO_3_. The metal concentration was determined by FAAS. The result obtained was quantitative and given in Table 6.

In order to check the validity of the proposed method, it was also applied to the water sample. A total of 25 mL of tap water, which was taken from Kütahya Dumlupınar University Analytical Chemistry Research Laboratory, and 0.3 μg/mL Cu(II) ions spiked into tap water sample, and then spiked and nonspiked samples were passed through the separate columns under optimum conditions. The samples have not been pretreated. After preconcentration, Cu(II) ions were determined by FAAS. Results were given in Table 6. According to the results, the method could be applied to water sample successfully.

### 3.3 Comparison with the literature results

The results obtained from the proposed method have been compared with the literature. As shown in Table 7, it can be clearly seen that the proposed method has higher adsorption capacity, selectivity, column reusability, preconcentration factor, and lower detection limit values.

## 4. Conclusion

Cu(II)-IAC material, which was synthesized by ion-imprinting technique, was successfully employed as a new solid-phase provides a selective, accurate, precise and fast method for the preconcentration and determination of copper ions. Also, to obtain a quantitative recovery for copper ions, there is no need for any chelating/complexing agent before the preconcentration procedure.

Characterization of the imprinting and nonimprinting sorbents was investigated using FTIR, SEM/EDX, and TG/DTA techniques. To evaluate the optimum parameters affecting the selective Cu(II) preconcentration, such as pH value, eluent type, sample flow rate, sample volume, capacity, and selectivity were investigated for both the ion-imprinted (Cu(II)-IAC) and the nonimprinted (Cu(II)-non-IAC) sorbents. The selectivity results showed that Cu(II) ion imprinting sorbent was highly selective even in the presence of other interfering ions. This finding is an indication that the synthesized adsorbent is advantageous. One of the main subjects of preconcentration/separation method development studies is a solid phase, which can be used easily and effectively. From this perspective, the ion-imprinted (Cu(II)-IAC) has better preconcentration factor, adsorption capacity, selectivity, low detection limit values, and also reusability of the sorbent were satisfactory when compared to literature. Especially, the detection limit, which was 0.03 μg/L, indicates an effective and sensitive determination of Cu(II) over other studies from literature. In addition, the preconcentration factor of the sorbent with a value of 150 is an indication that higher samples volumes can be studied easily. A total of 312.5 mg/g adsorption capacity value for the ion-imprinted (Cu(II)-IAC) is much higher than similar studies and a leading indicator for Cu(II) preconcentration/separation research. As a result, by this work, for Cu(II) preconcentration/separation, an effective and reusable solid phase has been brought to literature with superior specifications.

## Figures and Tables

**Table 1:** The effect of eluent type and concentration on the recovery of Cu(II) ions from the Cu(II)-IAC sorbent.

Eluent	Concentration of eluent (mol/L)	Recovery* %

HCl	0.5	73.0 ± 3.0
HCl	1.0	78.0 ± 1.0
HCl	2.0	91.0 ± 1.5
HNO_3_	0.5	92.0 ± 1.0

HNO_3_	1.0	94.0 ± 0.7

HNO_3_	2.0	95.4 ± 0.1

HNO_3_	4.0	100.8 ± 1.5

* N=3

**Table 2:** The selectivity parameters of the Cu(II)-IAC and Cu(II)-non-IAC sorbents.

Competitive ions	Sorbents	K_d_(Cu)	K_d_(X)	k	k′
Ni(II)	I	311.25	93.45	3.33	1.71
	II	327.70	167.67	1.95	
Mn(II)	I	8.22	3.64	2.26	2.13
	II	345.97	327.70	1.06	
Zn(II)	I	1561.25	270.49	5.77	3.95
	II	282.84	194.06	1.46	
Cd(II)	I	311.25	28.37	10.97	7.31
	II	259.17	172.36	1.50	
Pb(II)	I	259.17	35.51	7.30	19.73
	II	230.23	623.75	0.37	
Co(II)	I	1561.25	30.00	52.05	32.53
	II	248.75	155.00	1.60	
Cr(III)	I	479.52	45.05	10.65	2.31
	II	106.51	23.07	4.62	

I: Cu(II)-IAC

II: Cu(II)-non-IAC

**Table 3:** Calculated parameters from Langmuir and Freundlich isotherms for Cu(II)-IAC and Cu(II)-non-IAC sorbents.

Langmuir isotherm					Freundlich isotherm		
Sorbents	Q_m_ (mg/g)	K_L_ (L/mg)	R_L_	R^2^	n	K_F_	R^2^
Cu(II)-IAC	312.50	0.00239	0.943	0.956	1.57	2.95	0.972
Cu(II)-non-IAC	142.87	0.00254	0.283	0.882	1.67	1.90	0.954

**Table 4:** Analytical performance values.

Properties	Values
The instrumental detection limit (LOD), μg/L (N = 30)	4.51
The analytical detection limit, μg/L (LOD/PF)	0.038
The detection limit (LOQ), μg/L (N = 30)	13.52
The limit of quantification, μg/L (LOQ/PF)	0.11

**Table 5:** Precision values obtained from the proposed methods.

Variables	Values^a^
Average recovery efficiency	99.60
Standard deviation	± 0.048
RSD %	± 1.92
Relative error	–0.40 %
Number of measurements	7

a 95% confidence level

**Table 6:** Application of the proposed method to certified reference materials and real sample.

*Analytical results for Cu(II) determination in certified reference materials*			
Certified reference materials	Certified value (μg/mL )	Found (μg/mL)^a^	Recovery %
**Soft Drinking water ERML-CA021e**	1.98	1.90 ± 0.015	96.10
**NIST 1643e**	0.027 + 1.00 spike	0.93 ± 0.78	90.49
*Determination of Cu(II) in real samples*			
**Sample**	**Added (μg/mL)**	**Found (μg/mL)** a	**Recovery %**
**Tap water**	0	ND^b^	-
	1.50	1.470 ± 0.004	97.98

a N = 8.95% confidence level.

b Not detected.

**Table 7:** Comparison of the literature results of some different Cu(II)-imprinted solid phases.

Sorbent	Technique	Capacity (mg/g)	Sample volume (mL)	PF	LOD (μg/L)	Column regeneration	Selectivity	Ref.
Cu(II)-MTMAAm	FAAS	5.2	1000	100	0.90	10	Zn(II) 9.1 Ni(II) 14.8 Co(II) 26.6	[42]
Cu(II)-IIP	ICP-OES	II: 26.71 NI: 6.86	250	125	0.19	9	Zn(II)166.2 Ni(II)50.8 Co(II)72.3 Pb(II)175.8	[43]
Fe_3_O_4_@IIP-IDC	FAAS	174	250	50	1.03	-	Cd(II) 2.76 Pb(II) 4.38 Ni(II) 2.07 Zn(II) 7.81	[44]
Cu(II)-IMWCNT	HR-CS-AAS	II: 270.3 NI: 14.3	200	40	0.07	100	Ni(II)_0,5_ 5.42 Zn(II)_0,5_ 16.9	[46]
RAM–IIP	ICP-OES	15.9	15	30	0.17	40	-	[60]
Cu(II)-IAC	FAAS	II: 312.5 NI: 142.85	750	150	0.03	100	Ni(II) 1.70 Mn(II) 2.13 Zn(II) 3.95 Cd(II) 7.31 Pb(II) 19.73 Co(II) 32.53 Cr(III) 2.31	***This study***

IIP: ion imprinted polymer, RAM: restricted accessed material, PAR: MTMAAm: 5-methyl-2-thiozylmethacrylamide, II: ion imprinted, NI: non-imprinted, AC: Activated carbon, IMWCNT: imprinted multiwalled carbon nanotubes, IAC: imprinted activated carbon.
